# Biological Impact of Pd (II) Complexes: Synthesis, Spectral Characterization,* In Vitro* Anticancer, CT-DNA Binding, and Antioxidant Activities

**DOI:** 10.1155/2016/9245619

**Published:** 2016-02-16

**Authors:** Nitin Kumar Sharma, Rakesh Kumar Ameta, Man Singh

**Affiliations:** School of Chemical Sciences, Central University of Gujarat, Gandhinagar 382030, India

## Abstract

A new series of Pd (II) complexes of methyl substituted benzylamine ligands (BLs) has been synthesized and characterized via spectroscopic techniques such as UV/Vis. FTIR, LCMS, ^1^H, and ^13^C NMR. The UV/Vis study in DMSO, DMSO + water, and DMSO + PBS buffer (pH = 7.2) confirmed their molecular sustainability in liquids. Their* in vitro* anticancer activity against breast cancer cell lines such as MCF-7 and MDA-MB-231 makes them interesting for* in vivo* analysis. Their stronger DNA binding activity (DBA) compared with free ligand suggested them as a good DNA binder. DBA was further confirmed by physicochemical studies such as surface tension and viscosity of complex + DNA which inferred the disruption of DNA and intercalation of complexes, respectively. Their % binding activity, % disruption of DNA base pairs (DNABP), and % intercalating strength are reported in this paper for the first time for better understanding of DNA binding mechanism. Along with this, their scavenging activity (SA) determined through DPPH free radical and the results indicate good antioxidant behaviour of complexes.

## 1. Introduction

From the last few decades, the transition metal complexes with different amine ligands have drawn a significant interest in exploring anticancer activities, specifically related to solid tumor chemotherapies [1–4]. The breast cancer, a solid tumor, is one of the major issues in health care in terms of morbidity, mortality, and therapy costs [5]. To overcome these problems many more drugs have been introduced into the market but their response to therapy is still very poor. However, such drugs have not been found to be much effective for treatment of solid tumors due to their pertinent side effects such as nephrotoxicity, drug resistance, and renal and cervical problems [6, 7]. Therefore, the foremost target of most research groups is to find a convenient anticancer drug that can be used efficiently for the treatment of solid tumors. Recently, many Pd (II) complexes with promising anticancer activity against tumor cell lines have been synthesized and reported elsewhere [8]. In such studies, a good correlation was observed between the cytostatic activity and lipophilicity of the Pd complexes [6–8]. In fact, the Pd complexes, as a nonplatinum complex, have recently been reviewed to have a significant antitumor activity and lower side effects compared to cisplatin [5]. As an essential feature of metal-containing anticancer agents, Pd complexes are expected to have less kidney toxicity than cisplatin [7, 8]. Further many new Pd complexes with amine ligands having promising anticancer activities with lower side effect have recently been reported [5–12]. Bearing in mind that Pd (II) complexes are about 105 times more reactive than their Pt (II) analogues, the lower antitumor activity of Pd compounds has been attributed to very rapid hydrolysis of the leaving groups that dissociate readily in solution, leading to reactive species far from their pharmacological targets [9, 10].

Keeping above inconveniences, a new series of Pd (II) complexes has been synthesized with methyl substituted BLs and analyzed their* in vitro* antitumor activity on breast cancer cell lines MCF-7 and MDA-MB-231 which expressed effective anticancer potential. Since DNA is a primary molecular target of anticancer drugs and ascertains an extent of drug's chemotherapeutic potential, the DBA of synthesized complexes have chosen to investigate their anticancer nature [8, 13]. For better understanding of DBA, we have also calculated the % binding activity, % disruption of DNABP, and % intercalating strength of complexes by using newly developed quantitative equation. Apart from DNA binding, the antioxidant activity has also proven the anticancer nature and medicinal significance of the Pd (II) complexes which have been the criteria for studying their antioxidant activity [14–16]. Therefore, our study of Pd (II) complexes leads to a better understanding of their biological and medicinal applications.

## 2. Experimental Section

### 2.1. Materials and Methods

Palladium dichloride (PdCl_2_), benzylamine ligands (BLs), CT-DNA, Tris buffer, DMSO, and ethanol (>99.5%) were procured from Sigma-Aldrich and used as received. Elemental analysis was made with a Euro vector CHN analyzer and UV/Vis spectra were recorded with a Spectro 2060 plus spectrophotometer over 200–600 nm by using 1 cm path length cuvette. FTIR (Perkin Elmer) spectra were taken with KBr palate where polystyrene thin film was used as a calibration standard. ^1^H and ^13^C NMR spectra were recorded in DMSO-*d*_6_ (NMR, 99.99%) with a Bruker-Biospin Avance-III 500 MHz FT-NMR spectrometer. Mass spectra were obtained with PE SCIEX API 165 with +ve ESI mode with ammonium acetate and acetonitrile in 1 : 9 v/v ratio as mobile phase. Their molecular sustainability was determined in DMSO, DMSO/water, and DMSO/phosphate buffer of pH = 7.2. Buffer solution was prepared by adding 70 mL 0.1 M aqueous NaOH into 0.1 M aqueous KH_2_PO_4_ solution. The pH of a resultant buffer was checked with RS-232 modelled Cyber scan pH 2100, EUTECH pH meter.

### 2.2. General Consideration for Synthesis

Initially, PdCl_2_ and BLs were separately dissolved in freshly prepared aqueous ethanol (absolute ethanol and Milli-Q water in 1 : 1.5) in 1 : 2 molar ratio, using 1 MLH magnetic stirrer. The BLs solutions were added dropwise in metal compound solution with continuous stirring at room temperature. After 16 h, the mixture turned from light brown to greenish for Pd complexes and precipitates were formed. The ppts were filtered off and washed several times with chilled water/ethanol in 1 : 1 ratio and kept overnight in vacuum oven at room temperature for absolute dryness.

### 2.3. Characterization Data

#### 2.3.1. Complex 1: C_16_H_20_N_2_Pd [Pd2MBA]

Yield: 0.1810 g, 74.76%. Elemental analysis, found: C, 55.74; H, 5.26; N, 8.13%. Calcd for C_16_H_20_N_2_Pd: C, 55.82; H, 5.44; N, 8.22%. IR (KBr): *υ*_max_/cm^−1^ 3300 and 3214.2 (NH_2_), 1493 and 1449.7 (Ph, C=C), 740.7 (mono substituted Ph), 1051.9 (C–N), 479.6 (Pd–N). ^1^H NMR (125 MHz; DMSO-*d*_6_; Me_4_Si) *δ* 2.09 (2H, s, PhC**H**_2_NH_2_), 3.93 (2H, s, PhCH_2_N**H**_2_), 2.50 (3H. s, Ph**CH**_3_), 7.37–7.43 (1H, d, Ph**H**, *J* = 13.9 Hz), 7.45–7.47 (1H, d, Ph**H**, *J* = 7.3 Hz) and 7.35–7.38 (1H, m, Ph**H**). ^13^C NMR (125 MHz; DMSO-*d*_6_; Me_4_Si) *δ* 47.67 (C1), 136.52 (C2), 145.72 (C3), 129.99 (C4), 128.83 (C5), 125.89 (C6), 127.40 (C7) and 18.75 (C8). +ve ESI-MS:* m/z* 346.9 [M + 1] (calc. for [C_16_H_20_N_2_Pd] = 374.7). UV/Vis in DMSO: *λ*_max_ [*ε* (dm^3^mol^−1^cm^−1^)] = 275 (2569), 330 (664), 370 (477) nm, in DMSO: water (1 : 1): *λ*_max_ [*ε* (dm^3^mol^−1^cm^−1^)] = 270 (2921), 340 (358) nm, in DMSO: phosphate buffer (1 : 1): *λ*_max_ [*ε* (dm^3^mol^−1^cm^−1^)] = 265 (2252), 340 (410) nm.

#### 2.3.2. Complex 2: C_16_H_20_N_2_Pd [Pd3MBA]

Yield: 0.1722 g, 63.26%. Elemental analysis, found: C, 55.74; H, 5.26; N, 8.13%. Calcd for C_16_H_20_N_2_Pd: C, 55.79; H, 5.33; N, 8.19%. IR (KBr): *υ*_max_/cm^−1^ 3293 and 3210 (NH_2_), 1493 and 1449.7 (Ph, C=C), 744.64 (mono substituted Ph), 1099 (C–N), 437.42 (Pd–N). ^1^H NMR (500 MHz; DMSO-*d*_6_; Me_4_Si) *δ* 2.086 (2H, s, PhC**H**_2_NH_2_), 3.932 (2H, s, PhCH_2_N**H**_2_), 2.404 (3H, s, PhC**H**_3_), 7.21–7.23 (1H, d, Ph**H**, *J* = 7.2 Hz), 7.08–7.10 (1H, d, Ph**H**, *J* = 7.2) and 7.12–7.20 (1H, s, Ph**H**). ^13^C NMR (125 MHz; DMSO-*d*_6_; Me_4_Si) *δ* 47.674 (C1), 138.36 (C2), 137.23 (C3), 129.04 (C4), 127.90 (C5), 125.48 (C6), 128.15 (C7) and 20.98 (C8). +ve ESI-MS:* m/z* 346.05 [M + 1] (calc. for [C_16_H_20_N_2_Pd] = 344.75). UV/Vis in DMSO: *λ*_max_ [*ε* (dm^3^mol^−1^cm^−1^)] = 275 (2509), 340 (595), 370 (486) nm, in DMSO: water (1 : 1): *λ*_max_ [*ε* (dm^3^mol^−1^cm^−1^)] = 270 (2745), 340 (344) nm, in DMSO: phosphate buffer (1 : 1): *λ*_max_ [*ε* (dm^3^mol^−1^cm^−1^)] = 265 (2086), 340 (412) nm.

#### 2.3.3. Complex 3: C_16_H_20_N_2_Pd [Pd4MBA]

Yield: 0.1785 g, 74.20%. Elemental analysis, found: C, 55.74; H, 5.26; N, 8.13%. Calcd for C_16_H_20_N_2_Pd: C, 55.88; H, 5.29; N, 8.21%. IR (KBr): *υ*_max_/cm^−1^ 3257 and 3210 (NH_2_), 1453 and 1355 (Ph, C=C), 756.46 (mono substituted Ph), 1000.7 and 1036.1 (C–N), 492.6 (Pd–N). ^1^H NMR (500 MHz; DMSO-*d*_6_; Me_4_Si) *δ* 2.09 (2H, s, PhC**H**_2_NH_2_), 3.93 (2H, s, PhCH_2_N**H**_2_), 2.31 (3H, s, PhC**H**_3_), 7.33–7.35 (1H, d, Ph**H**, *J* = 6.0 Hz), 7.18–7.20 (1H, d, Ph**H**, *J* = 10.72 Hz) and 7.25 (1H, s, Ph**H**). ^13^C NMR (125 MHz; DMSO-*d*_6_; Me_4_Si) *δ* 47.67 (C1), 139.11 (C2), 127.89 (C3), 128.76 (C4), 138.23 (C5), 129.11 (C6), 128.14 (C7) and 20.29 (C8). +ve ESI-MS:* m/z* 346.9 [M + 1] (calc. for [C_16_H_20_N_2_Pd] = 344.75). UV/Vis in DMSO: *λ*_max_ [*ε* (dm^3^mol^−1^cm^−1^)] = 280 (2208), 340 (447), 370 (368) nm, in DMSO: water (1 : 1): *λ*_max_ [*ε* (dm^3^mol^−1^cm^−1^)] = 265 (2770), 340 (286) nm, in DMSO: phosphate buffer (1 : 1): *λ*_max_ [*ε* (dm^3^mol^−1^cm^−1^)] = 265 (2276), 340 (371) nm.

## 3. Biological Evaluation

### 3.1. *In Vitro* Anticancer Activity

Cell viability was estimated colorimetrically using 2-(3-diethylamino-6 diethylazaniumylidene-xanthen-9-yl)-5-sulfobenzenesulfonate, SRB (sulforhodamine B) as standard assay with high reproducibility [17].

#### 3.1.1. Cell Lines and Culture Conditions

Human breast cancer cell lines MCF-7 and MDA-MB-231 were obtained from NCI, USA, and grown in minimal essential medium (MEM). Eagles media were supplemented with 10% heat inactivated fetal bovine serum (FBS, Sigma-Aldrich), 2 mM L-glutamine, and 1 mm sodium pyruvate (Hyclone) in humidified CO_2_ incubator.

#### 3.1.2. Assay of Cytotoxicity in Cancer Cell Lines

Cytotoxicity of Pd (II) complexes was determined by SRB assay where E5000 cells were seeded into each well of a 96-well clear flat bottom polystyrene tissue culture plate and incubated for 2 h in MEM. An additional 190 mL cell suspension was added in each well containing 10 mL test sample in 10% DMSO with 10 mL Adriamycin (doxorubicin) as a positive drug control. Each experiment was carried out in 3 replicate wells. After an incubation of 48 h, 100 mL of 0.057% SRB solution (w/v) was added in each well. Then 200 mL of 10 mM Tris base solution (pH = 10.5) was added into each well and shaken smoothly. The cell viability was assayed by absorption at 510 nm with a microplate reader. The experiments were repeated thrice with 3 replicates each time and 99% reproducibility was obtained.

### 3.2. DNA Binding

CT-DNA (analytical grade, Sigma-Aldrich) was used as received. Tris-HCl buffer (10 mM, pH = 7.2) was prepared in Milli-Q water for 50 *µ*M DNA stock solution and used for absorption titration, viscosity, surface tension, conductivity, and zeta potential measurements.

#### 3.2.1. Absorption Spectroscopy

DNA concentration was determined using an absorption spectrophotometer that gave 6600 M^−1^cm^−1^, a molar absorption coefficient at 260 nm [18, 19]. The CT-DNA in buffer gave a ratio of UV absorbance at 260 and 280 nm which is found to be 1.8 to 1.9 and indicates a presence of protein free DNA [20–22]. The 10, 30, 50, 70, and 90 *µ*M complex solutions were prepared in 10% DMSO in Tris buffer for Pd(BLs)_2_ to which the DNA stock solutions were added. Their ri = [complex]/[DNA] at 0.2, 0.6, 1, 1.4, and 1.8 were calculated by absorption titration. Before recording UV spectra the Pd(BLs)_2_ + DNA solutions were incubated for 15 min at rt to enable sufficient and reproducible DNA binding with complexes. Further their binding strength, an intrinsic binding constant (*K*_*b*_) with CT-DNA, was obtained by monitoring a change in DNA absorbance on increasing Pd(BLs)_2_ concentrations and calculated with the following equation [23]:

(1) $$\begin{array}{c} \frac{\left[DNA\right]}{\varepsilon_{a}-\varepsilon_{b}}=\frac{\left[DNA\right]}{\varepsilon_{b}-\varepsilon_{f}}+\frac{1}{K_{b}\left(\varepsilon_{b}-\varepsilon_{f}\right)}. \end{array}$$

*ε*_*a*_, *ε*_*f*_, and *ε*_*b*_ are apparent, free, and bound complex extinction coefficients, respectively. *ε*_*f*_ was determined from a calibration curve of an isolated metal complex, with Beer-Lambert law. *ε*_*a*_ was determined as a ratio of the measured absorbance and Pd (II) complexes concentration similar to *A*_obs_/[Pt]. A plot of [DNA]/(*ε*_*a*_ − *ε*_*f*_) versus [complex] produced a slope of 1/(*ε*_*b*_ − *ε*_*f*_) and a *Y* intercept equal to 1/*K*_*b*_(*ε*_*b*_ − *ε*_*f*_); *K*_*b*_ is the ratio of slope to the *Y* intercept [23].

#### 3.2.2. Physicochemical Analysis

(*1) Viscosity and Surface Tension*. Viscosity and surface tension measurements were made using BMS at 298.15 K and the temperature controlled through an auto temperature control LAUDA ALPHA RA 8 thermostat [24, 25]. About 15 to 20 measurements were made for each composition, enabling high reproducibility and precision, from which viscous flow time (VFT) and pendent drop numbers (PDN) were calculated. (*η*/*η* ^0^)^1/3^ versus binding ratio was calculated where *η* represents the dynamic viscosity of DNA with complexes while *η* ^0^ is the viscosity of a DNA mixture in buffer.

(*2) Conductance and Zeta Potential*. Conductance and zeta potentials of DNA solutions with and without complexes were measured using LABINDIA, PICO + conductivity and Microtrac Zetatrac, U2771, and DLS, respectively, at 25°C. Aqueous KCl at 0.1, 0.01, and 0.001 M of 12.88, 1.413, and 147 mScm^−1^, respectively, were used for calibration of conductivity meter. Similarly, an auto suspended solution of alumina suspension (400-206-100) was used as zeta potential standard. Initially, a set-zero was made with DMSO + Tris buffer for Pd(BLs)_2_. The DNA concentration for all measurements was kept constant while the concentration of Pd(BLs)_2_ was varied from 10 to 90 *µ*M over 20 *µ*M intervals.

### 3.3. Scavenging Activities

Antioxidant activities have been studied on free radical scavenging of stable 1-2,5-diphenyl-2-picrylhydrazyl (DPPH^•^) [26]. For this purpose stock solution of complexes and DPPH^•^ (0.002%) were mixed in DMSO + water (1 : 1) for Pd(BLs)_2_. For sample preparation, the DPPH^•^ solution was mixed with a complex solution in 1 : 1 ratio followed by vigorous shaking and thereafter kept in dark for 30 min incubation. The UV absorbance was measured at 517 nm with UV/Vis spectrophotometer. Later, the radical scavenging activity was measured and a decrease in DPPH absorbance indicated a radical scavenging activity, calculated with the following equation:

(2) $$\begin{array}{c} \text{Scavenging activity}\%=\left(\;\frac{A_{0}-A_{s}}{A_{0}}\;\right)\times 100. \end{array}$$

The *A*_*s*_ is absorbance of DPPH^•^ with a test compound and *A*_0_ absorbance of DPPH^•^ without a test compound. Absorbance data are presented as means ± SD of three determinations.

## 4. Results and Discussion

### 4.1. Synthesis and Characterizations

**Figure 1:**
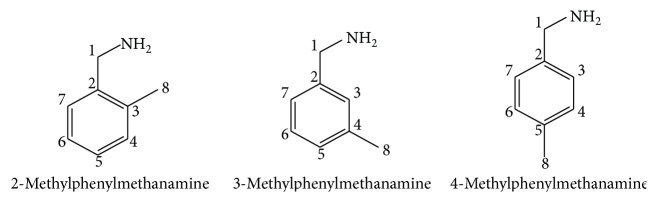
Structures of the ligands (L = BLs).

Initially, the PdCl_2_ and BLs ligands were mixed in 1 : 2 molar ratio for the synthesis of Pd(BLs)_2_ complexes (Figure 1). The reaction was conducted in aqueous ethanol solution for 14 to 16 h as per reaction scheme given below.

*Synthesis of Pd (II) Complexes.* Consider

(3) $$\begin{array}{c} {PdCl}_{2}+2BLs\overset{Aqueous\;\;C_{2}H_{5}OH}{\underset{16\;h/RT}{\to }}Pd{\left(\;BLs\;\right)}_{2} \end{array}$$

**Figure 2:**
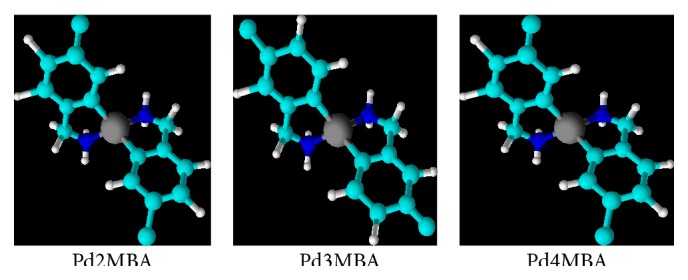
Structure of synthesized Pd (II) complexes.

**Figure 3:**
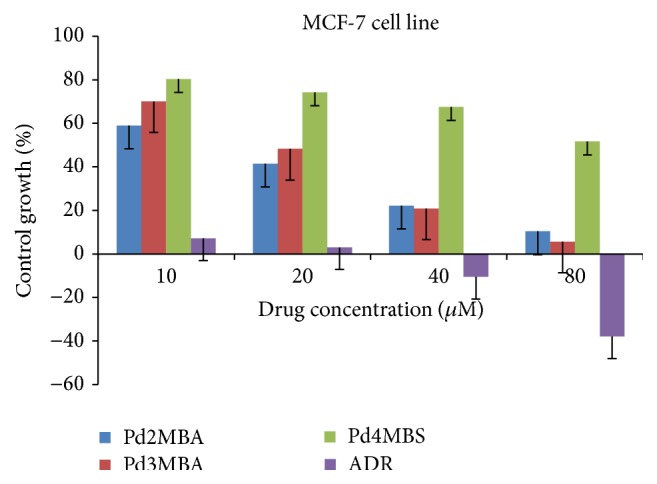
Growth curve: human breast cancer cell line (MCF-7).

The 3300 to 3119 cm^−1^ stretching frequencies inferred presence of –NH_2_ of benzylamine in the Pd(BLs)_2_ and similarly from 1497 to 1453 cm^−1^ predicted C=C in phenyl ring. The 495.92 to 438.78 cm^−1^ and 380–348 cm^−1^ indicates Pd–N coordinate and Pd–Cl bands, respectively [26, 27]. In ^1^H NMR, the 2H of –N**H**_2_ and PhC**H**_2_– appeared at *δ* 3.93 to 2.09 with singlet for Pd(BLs)_2_. The PhC**H**_3_ proton of benzylamine appeared at *δ* 2.50 to 2.31 with singlet for all Pd(BLs)_2_ [28]. Two doublets at *δ* 7.37–7.42 and 7.49–7.47 having *J* = 13.9 and 7.3 MHz, respectively, in Pd2MBA for H of C4 and C6 appeared. Similarly, a singlet for 1H of C3 at *δ* 7.20 value and doublets for 1H of C5 and C6 at *δ* 7.08–7.10 and 7.21–7.23 (*J* = 7.2 MHz), respectively, were found for Pd3MBA. A doublet at *δ* 7.33–7.35 and 7.18–7.20 with *J* = 6.0 and 10.72 MHz, respectively, was confirmed for 1H of C3 and C4 positions while a singlet was confirmed for 1H of C6 at *δ* 7.25 in Pd4MBA. In ^13^C NMR, the benzyl carbon (Ph**C**H_2_–) appears at *δ* 47.66 for all the Pd(BLs)_2_ [28]. The aromatic ortho, meta, and para –CH_3_ attached carbon appeared at *δ* 145.72, 129.036, and 138.23, respectively, for the Pd(BLs)_2_. The carbon of –CH_3_ at ortho, meta, and para appeared within *δ* 20.98 to 18.72 ppm. The +*ve* ESI mass spectra of Pd(BLs)_2_ have found [M + 1] confirming their molecular mass. In UV study, the absorption spectrum consists of a band at 400 nm and may be assigned as 1A_1g_ → 1A_2g_ (*d*_*xy*_ → *d*_*x*^2^−*y*^2^_) transition occurring with Pd complexes (see ESI, Figures 1–3, in Supplementary Material available online at http://dx.doi.org/10.1155/2016/9245619) [29]. To investigate their molecular stability in solution, the UV/Vis spectral behaviour was investigated in DMSO, DMSO + water, and DMSO + phosphate buffer for Pd(BLs)_2_ (ESI, Figures 1–3). The overall patterns of spectra for complexes solution were found to be similar to the different mediums to ensure their stability. Thus our synthesized Pd (II) complexes having UV/Vis absorption from 265 to 270 nm and ^1^H NMR coupling constant between 5 and 10 MHz have confirmed their trans geometry (Figure 2).

### 4.2. Anticancer Activity

**Figure 4:**
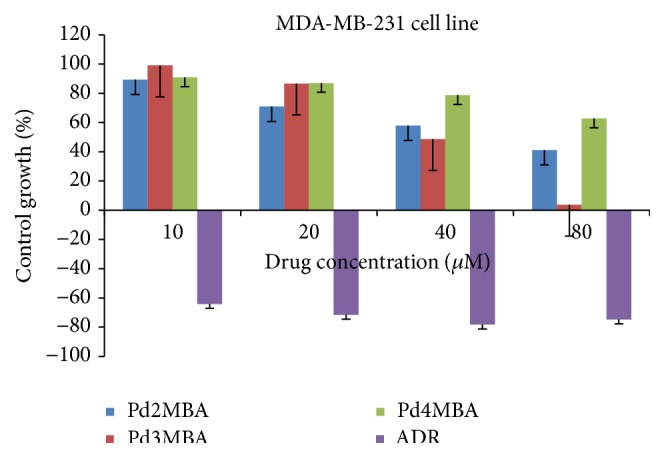
Growth curve: human breast cancer cell line (MDA-MB-231).

Moving towards the higher anticancer potential of Pd (II) complexes, many of the* in vitro *studies on different cell lines have been reported, suggesting their cytotoxicity up to some extent [30–33]. In such studies authors have synthesized some Pd complexes with different amine ligands and report their cytotoxicity when tested* in vitro* [30–33]. It seems that these amine ligands and their derivatives can further improve the cytotoxicities when they reacted with different metals. Therefore, in our study we tried to focus on the factors, namely, nature of ligands and methyl group substitution on ligand, which can modulate the cytotoxicity of such complexes. In this context, we have demonstrated an* in vitro* anticancer study of some novel Pd complexes using BLs as ligands for better anticancer activity. The complexes were tested* in vitro* against MCF-7 and MDA-MB-231 human breast tumor cell lines by colorimetric microculture 2-(3-diethylamino-6-diethylazaniumylidene-xanthen-9-yl)-5-sulfobenzenesulfonate (SRB) assay and compared with Adriamycin (ADR) and cisplatin [6, 34]. The MCF-7 and MDA-MB-231 cell lines anticancer activities for 10, 20, 40, and 80 *µ*M Pd(BLs)_2_ have been illustrated in Figures 3 and 4. The analyzed GI_50_, TGI, and LC_50_ in *µ*M have inferred 50% growth inhibition, resultant total growth inhibition, and a net loss of 50% cells after treatment, respectively (Table 1).

The GI_50_ value less than 10 *µ*M depicts the anticancer activity with respect to ADR and cisplatin, where the closer GI_50_ values of Pd2MBA, Pd3MBA, and Pd4MBA from standards make them interesting for testing against other cancerous cell lines.

### 4.3. DNA Binding Activity

#### 4.3.1. Spectrophotometric Method

**Figure 5:**
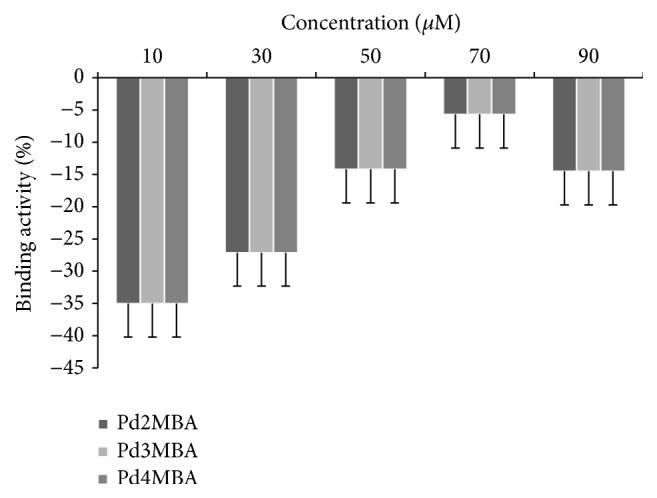
% binding affinity of complexes at 260 nm.

**Figure 6:**
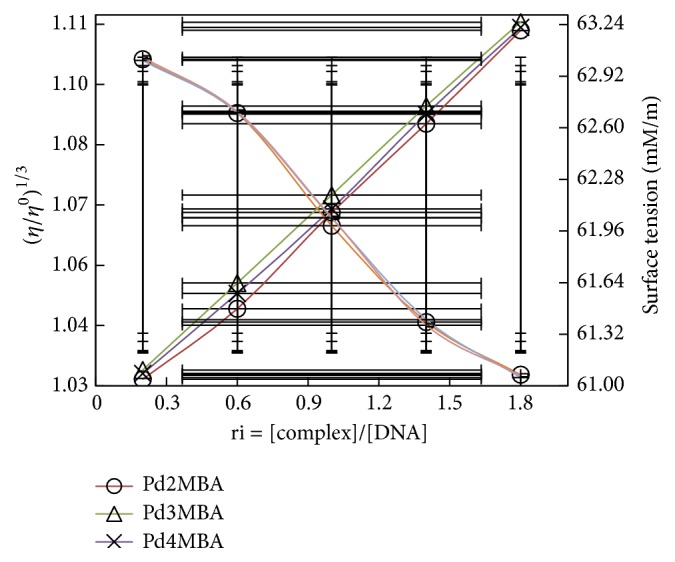
Effect of increasing amounts of Pd based benzylamine complexes on viscosity and surface tension of CT-DNA (5 × 10^−5^ M).

The DBA explains the anticancer nature of the complex or the drug and can be analyzed by the absorption spectral study. In light of this, the Pd (II) complexes were mixed with CT-DNA at certain concentration and shown the changes in absorbance. Generally, the hypochromism effect in UV study reveals all synthesized complexes intercalative binding strength attributed to an interaction with DNA bases [35, 36]. Similarly, a hyperchromic effect ascribes an external contact or a partial uncoiling of DNA structure; exposing more bases of DNA may be due to electrostatic binding [37, 38]. Such observations have been noticed in the present study where a significant hypochromic effect in absorption titrations of DNA with Pd (II) exposed an intercalation with the base pairs of DNA [37, 38] (ESI, Figures 4–6).

**Figure 7:**
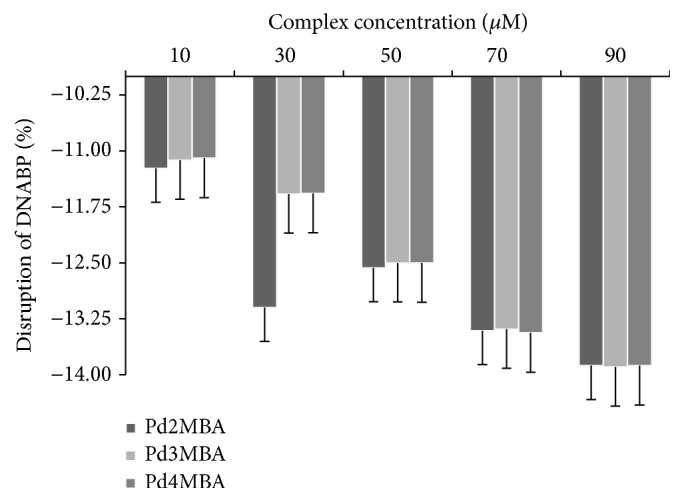
% disruption of DNA base pairs interaction.

The decrease in UV absorption predicted the strong interactions of Pd2MBA, Pd3MBA, and Pd4MBA, whose intercalating strengths depend on size and electron densities of interacting aromatic rings with an amine group [35–38]. Their DBA has been found stronger in comparison to a free ligand like benzylamine (ESI, Figure 7).

*Comparative % Binding Affinity of Complexes*. For a better understanding of their binding potential, a % binding affinity (BA) with DNA has been calculated through well known spectrophotometric method at a characteristic wavelength of 260 nm. The % BA was calculated as per the following equation (Figure 5):

(4) $$\begin{array}{c} \%\text{ BA of Complexes}=\frac{Absorbance\;\;of\;\;complex\;\;at\;\;260\;nm-Absorbance\;\;of\;\;DNA\;\;at\;\;260\;nm}{Absorbance\;\;of\;\;DNA\;\;at\;\;260\;nm}\times 100. \end{array}$$

Figure 4 shows the BA of each complex where negative sign explains their strong binding with DNA and hypochromism of the complexes.

The all Pd (II) complexes show approximately the same binding activity at each concentration indicating the same binding potential of each complexes. The same binding potential can further be explained by the same geometry of the all complexes. Therefore, it can be understood that the Pd complexes with the same geometry may equally participate when they interacted with DNA.

#### 4.3.2. Physicochemical Method

Along with spectrophotometric study, the physicochemical study, such as viscosity, surface tension, conductivity, and zeta potentials, is also an effective method for better understanding of DBA. Thus, we have mathematically established a comparison of their physicochemical data that presumes their binding affinity with DNA at structural level.

(*1) Study of Structural Disruption of CT-DNA*. Initially, the complex entertains the DNA and attacks on its base pairs; therefore it weakens the intermolecular forces of bases which results in the disruption of base pairs. Since, it is an intermolecular force disruption and such disruption can be measured by the analysis of surface tension, therefore, we have analysed and compared the surface tensions of DNA with and without complexes. Generally, the decrease in surface tension infers the force disruption and we have also obtained such results where we compared the surface tension of pure DNA and DNA + complexes. For instance, the surface tension values of the DNA solution with increasing amounts of Pd (II) complexes (1/*R* = 0.2, 0.6, 1.0, 1.4, 1.8) have decreased as compared to pure DNA (Figure 6).

A decrease in surface tension of Pd (II) complexes + DNA inferred the weakening of intermolecular interaction of DNA bases and has lost interaction with complexes.

*Complex Contributions on Disruption of DNA Base Pairs*. Surface tension is an important analysing aspect for the disruption of DNA base pairs; therefore, for the advanced study we have also measured and compared the % disruption of DNA base pairs. The % complex contribution was calculated on the disruption of DNA (Figure 7):

(5) %  disruption  of  DNA  base  pairs=γ  of  DNA  with  complexes−γ  of  pure  DNAγ  of  pure  DNA×100.

Figure 7 shows the disruption of DNABP with respect to complexes and with negative values indicating the weakening interaction of DNA bases towards disruption. This negative values support the binding affinity (BA) of complexes where the lower –*ve* values of Pd complexes indicate the disruption of DNABP.

(*2) Study on Intercalation Phenomenon of Complexes*. For the classical intercalative mode, the viscosity of the DNA solution is increased due to an increase in overall DNA length while it is decreased for a partial nonclassical intercalation process causing a bend or kink in the DNA helix that reduces its effective length concomitantly [39, 40]. For instance, a decrease in relative viscosity with cisplatin is explained due to covalent binding and shortening in an axial length of double helix DNA. On the other hand, classical organic intercalator such as ethidium bromide increased the relative viscosity with increasing of an axial length of the DNA [41–43]. To observe covalent binding or classical organic interaction, their relative specific viscosities (*η*/*η* ^0^)^1/3^ (*η* ^0^ and *η* being specific viscosity contributions of DNA with and without complexes, resp.) were plotted against 1/*R* (*R* = [DNA]/[complex] = 0.2, 0.6, 1.0, 1.4, 1.8) (Figure 6).

On increasing concentration of Pd(BLs)_2_, an increase in viscosity of DNA was observed, proving the Pd(BLs)_2_ as DNA intercalators (Figure 6). For ethidium bromide, the relative viscosity of DNA is increased with a slope from 0 to 0.9448 L/mM [44], whereas with 0.001 the relative viscosity of DNA has increased for Pd2MBA, Pd3MBA, and Pd4MBA. The viscosity of free ligand (2-methylbenzylamine) with DNA was measured and found lower than the complexes + DNA, which inferred that interaction of DNA with free ligand is weaker as compared to complex. Therefore, a higher increase in viscosity of Pd complex + DNA as compared to free ligands clearly explained the stronger intercalation between complexes and DNA [42]. The constant value of the slope and increasing values of viscosity infer almost similar intercalating nature for all the complexes.

*Complex Contributions towards Intercalation with DNA Base Pairs*. Apart from the above discussion, we have also established the % binding affinity with respect to viscosity (*η*) using two different methods. In both methods the % complex on the intercalation with DNA was calculated:

(6) %Intercalating  strength  of  complexes=η  of  DNA  with  complexes−η  of  pure  DNAη  of  pure  DNA×100.

**Figure 8:**
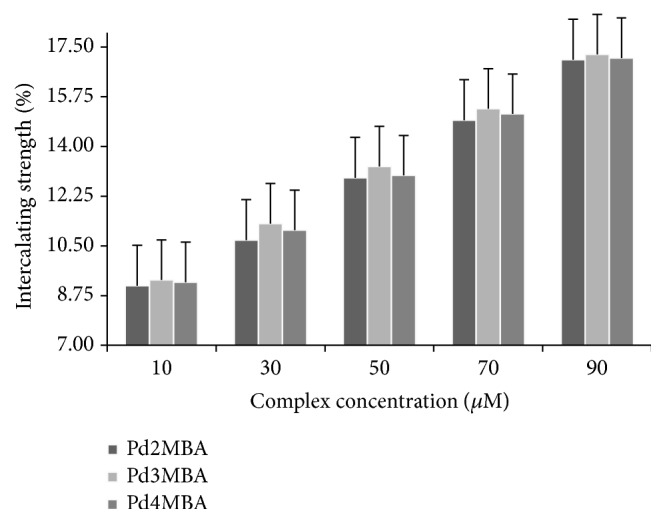
% intercalating strength of complexes with DNA base pairs.

**Figure 9:**
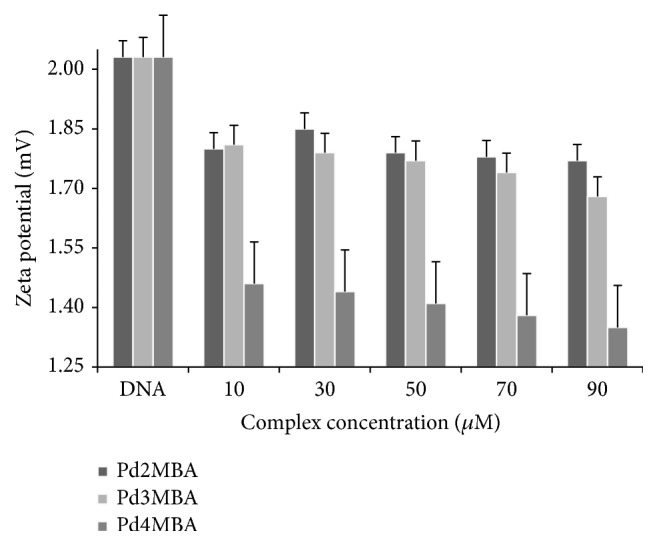
Zeta potential (mV) of pure DNA and DNA + complexes.

Figure 8 clearly explains the individual BA of complexes with DNA base pairs (DNABP). The Pd2MBA, Pd3MBA, and Pd4MBA expressed lower BA when they interacted with DNA.

(*3) Conductivity and Zeta Potential*. The DNA and complexes are charge molecules; therefore their conductivity and zeta potential have also been considered as interaction parameters as well as key factors for an analyzing conformational behaviour of an isolated DNA chain with the complexes. A DNA solution of 50 *µ*M had 3426 *µ*S conductivity while, with increasing amounts of complexes, the decrease in zeta potential as well as conductivities has been found (Figure 9 and Table 2). DNA molecules are negatively charged due to phosphate groups, but due to interaction with complexes their negative charge density is decreased due to Columbic interaction of positively charged complexes and the disassociation of counter ions in DNA.

The decrease in conductivity and zeta potential confirmed that the DNA could structurally be modified due to such interactions.

### 4.4. Drug Efficacy Studies or DFI with DNA Binding

For the better understanding of Pd(BLs)_2_ + DNA interaction and their disruption during an intercalation process, DFI has found reliable data in support of anticancer activity [42]. Therefore, our study infers that the Pd(BLs)_2_ + DNA interactions at different concentration have shown their higher viscosities and lower surface tension with respect to DNA solution (Figure 6). Thus, a disruption of DNABP decreases surface tension by developing an interaction between complexes and DNA.

### 4.5. Scavenging Activities

**Figure 10:**
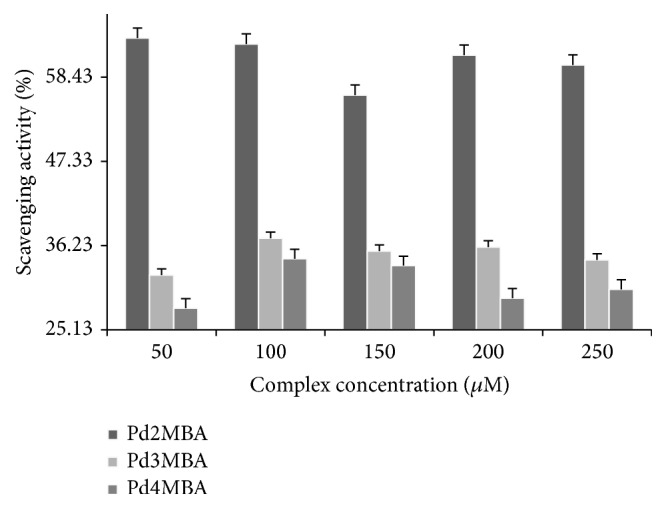
Free radical scavenging activities of synthesized complexes.

The scavenging activities have been investigated to support the anticancer potential of such complexes [43, 44]. Recently it is reported that, by using hydroxyl radicals, an inorganic radical (most dangerous reactive oxygen species) with its precursor such as H_2_O_2_ proposed the facts responsible for their mode of mechanism [43]. On the basis of the above study, the antioxidant activities have studied and analyzed the decrease in absorbance or scavenging effect of a stable free DPPH^•^ as per standard procedure for the Pd(BLs)_2_ [45, 46]. The percentage scavenging activity of Pd(BLs)_2_ has been determined in a concentration-dependent mode in comparison with the DPPH^•^ absorption at 517 nm [47–50]. The DPPH^•^ free radical's absorption at 517 nm with DMSO was 0.906 and for Pd(BLs)_2_. From 50 to 250 *µ*M with an interval of 50 *µ*M, complexes have expressed a decrease in absorption (Figure 10) that characterised them as an antioxidant [51].

The 55.47, 28.812, and 24.24% for Pd2MBA, Pd3MBA, and Pd4MBA, respectively, inferred Pd2MBA, a strong antioxidant among them. Thus, the antioxidant activities of Pd complexes have inferred their significance in medicinal sciences.

## 5. Conclusion

Since the synthesized Pd(BLs)_2_ could not express the anticancer activity, their DBA and SA refer them as anticancer compounds having some modification. The closer GI_50_ values as compared to control drugs such as doxorubicin and cisplatin make the synthesized complexes more interesting to test against other cancerous cell lines. The DBA analysis with viscosity, surface tension, zeta potential, and conductance has inferred strong intercalating as well as DNA disruption and intercalating nature of the complexes. The quantitative evaluation of % DNABP disruption and % intercalating strength advances the DNA binding mechanism. The complexes have shown significant free radical scavenging activities acting as antioxidants and could be used for medicinal purposes. Thereby, our study could have potential in better understanding of medicinal aspects of the complexes.

## Supplementary Material

The supplementary material contains UV/Vis spectra of complexes in different solvent and spectra of DNA binding with and without complexes.

## Figures and Tables

**Table 1:** LC_50_, TGI, and GI_50_ values (*µ*g/mL) against MCF7 and MDA-MB-231 cell lines of complexes, an anticancer analysis.

Entry	Complexes	Drug concentrations (*µ*M) calculated from graph					
		MCF7			MDA-MB-231		
		LC_50_	TGI	GI_50_	LC_50_	TGI	GI_50_
1	Pd2MBA	>80	79.4	27.5	>80	>80	61.5
2	Pd3MBA	>80	75.6	30.1	>80	>80	44.6
3	Pd4MBA	>80	>80	78.4	>80	>80	>80
4	ADR	79.2	40.5	<10	39.85	<10	<10
5	Cisplatin	>30	>30	<10	>30	>30	<10

**Table 2:** Conductivity (*µ*S) of DNA and DNA + complexes.

Conductivity/*µ*S						
	0.00^*∗*^	10	30	50	70	90
Pd2MBA	3426	3053	3013	2974	2903	2898
Pd3MBA	3426	2988	2978	2966	2952	2912
Pd3MBA	3426	2761	2687	2645	2581	2555

*∗* refers to  50 *µ*M DNA.

## Supporting Information

It includes UV/Vis spectra of stability, DNA binding, and conductivities (DNA + complexes) of the complexes.
