# Supplementary material for: Biological Impact of Pd (II) Complexes: Synthesis, Spectral Characterization, In Vitro Anticancer, CT-DNA Binding, and Antioxidant Activities
Source: Int J Med Chem. 2016 Feb 16;2016:9245619. doi: 10.1155/2016/9245619 (PMC4771903; doi:10.1155/2016/9245619)
Supplement: Supplementary file 1 — The supplementary material contains UV/Vis spectra of complexes in different solvent and spectra of DNA binding with and without complexes. [file 9245619.f1.docx]

**Electronic Supplementary Information (ESI)**

**Figure’s Caption**

Figure1. UV Spectra of Pd (II) complexes in DMSO at 0.001M

Figure 2. UV Spectra of Pd (II) complexes in DMSO-Water

Figure 3. UV Spectra of Pd (II) complexes in DMSO-Phosphate buffer at 7.2 pH

Figure 4. Absorption spectra of DNA (5x10ˉ^5^M) in absence and presence of increasing amounts of Pd2MBA (ri = [complex]/[DNA]).

Figure 5. Absorption spectra of DNA (5x10ˉ^5^M) in absence and presence of increasing amounts of Pd3MBA (ri = [complex]/[DNA]).

Figure 6. Absorption spectra of DNA (5x10ˉ^5^M) in absence and presence of increasing amounts of Pd4MBA (ri = [complex]/[DNA]).

Figure 7. Absorption spectra of DNA (5˟10^-5^M) in absence and presence of increasing amounts of benzylamine (ri = [complex]/[DNA]).

**Figure 1.**

**Figure 2.**

**Figure 3.**

**Figure 4.**

**Figure 5.**

**Figure 6.**

**Figure 7.**
